# A Comparative Study on *In Vitro* Osteogenic Priming Potential of Electron Spun Scaffold PLLA/HA/Col, PLLA/HA, and PLLA/Col for Tissue Engineering Application

**DOI:** 10.1371/journal.pone.0104389

**Published:** 2014-08-20

**Authors:** Hanumantha Rao Balaji Raghavendran, Subramaniam Puvaneswary, Sepehr Talebian, Malliga Raman Murali, Sangeetha Vasudevaraj Naveen, G. Krishnamurithy, Robert McKean, Tunku Kamarul

**Affiliations:** 1 Tissue Engineering Group (TEG), National Orthopaedic Centre of Excellence in Research and Learning (NOCERAL), Department of Orthopaedic Surgery, Faculty of Medicine, University of Malaya, Kuala Lumpur, Malaysia; 2 The Electrospinning Company Ltd, Rutherford Appleton Laboratory, Harwell Oxford, Didcot, Oxfordshire, United Kingdom; 3 Clinical Investigative Centre, Faculty of Medicine, University Malaya Medical Center, Kuala Lumpur, Malaysia; 4 Department of Mechanical Engineering, Engineering Faculty, University of Malaya, Kuala Lumpur, Malaysia; University Hospital of the Albert-Ludwigs-University Freiburg, Germany

## Abstract

A comparative study on the in vitro osteogenic potential of electrospun poly-L-lactide/hydroxyapatite/collagen (PLLA/HA/Col, PLLA/HA, and PLLA/Col) scaffolds was conducted. The morphology, chemical composition, and surface roughness of the fibrous scaffolds were examined. Furthermore, cell attachment, distribution, morphology, mineralization, extracellular matrix protein localization, and gene expression of human mesenchymal stromal cells (hMSCs) differentiated on the fibrous scaffolds PLLA/Col/HA, PLLA/Col, and PLLA/HA were also analyzed. The electrospun scaffolds with a diameter of 200–950 nm demonstrated well-formed interconnected fibrous network structure, which supported the growth of hMSCs. When compared with PLLA/H%A and PLLA/Col scaffolds, PLLA/Col/HA scaffolds presented a higher density of viable cells and significant upregulation of genes associated with osteogenic lineage, which were achieved without the use of specific medium or growth factors. These results were supported by the elevated levels of calcium, osteocalcin, and mineralization (P<0.05) observed at different time points (0, 7, 14, and 21 days). Furthermore, electron microscopic observations and fibronectin localization revealed that PLLA/Col/HA scaffolds exhibited superior osteoinductivity, when compared with PLLA/Col or PLLA/HA scaffolds. These findings indicated that the fibrous structure and synergistic action of Col and nano-HA with high-molecular-weight PLLA played a vital role in inducing osteogenic differentiation of hMSCs. The data obtained in this study demonstrated that the developed fibrous PLLA/Col/HA biocomposite scaffold may be supportive for stem cell based therapies for bone repair, when compared with the other two scaffolds.

## Introduction

Biomaterials are revolutionizing many aspects of preventive and therapeutic healthcare, having roles in areas such as tissue engineering [1]. Currently, they have an important role in the development of new medical devices, prostheses, tissue repair, drug delivery systems, and diagnostic techniques [2]. Substantial efforts have been taken in the field of tissue engineering to develop potential biocomposite scaffold for biomedical applications by utilizing electrospinning approach. An ideal biocomposite scaffold should mimic bone extra cellular matrix (ECM) in terms of chemical composition and physiological environment in order for the cells to get attached, proliferate, and differentiate preferably without supplementation of specific growth factors, which is best demonstrated using in vivo models [3]. However, for this material to be tested in vivo, its matrix must be biologically compatible. It has been demonstrated that the use of collagen scaffold may be advantageous because collagen is found in abundance in many tissues and organs of the living organisms [4]. However, as a naturally derived material, collagen scaffolds have been reported to possess immunogenicity and contain pathogenic impurities. In addition, control over their mechanical properties and degradability appears to vary. On the other hand, other biodegradable synthetic polymers, such as poly L-lactic acid (PLLA), could be a good choice as the base for producing scaffolds using electrospinning techniques, because they elicit low immunogenic response [5]. It has been reported that electrospun fibers developed using collagen exhibit osteoconductivity and osteoinductivity in tissue engineering applications. A major advantage of PLLA over collagen is that it can be produced on a large scale with controlled strength, degradation, and micro or nanostructure with great flexibility, thus meeting specific demands. However, the disadvantage of such synthetic polymers is that it has poor biocompatibility and results in the release of acidic degradation products that may alter the physiological environment, thus affecting cellular functions [6]. Therefore, a combination of biomaterials, such as collagen and HA, in appropriate ratio with PLLA to form a biocompatible scaffold could be a better strategy to overcome some major concerns such as compatibility and cellular differentiation of multipotent stem cells.

The methods of co-spinning of collagen I (Col) with synthetic polymer and coating the Col on the polymer fibers to produce optimized biological functions have been previously demonstrated [7]. It has been reported that PLLA-based scaffolds with collagen and HA cultured with human fetal osteoblasts (hFOBs) on nanofibers produced increased osteogenic markers than PLLA/HA scaffolds after 20 days with osteogenic media supplements. Furthermore, the nodule formation by hFOB cells cultured on PLLA/Col/HA scaffolds was higher than that on PLLA/HA scaffolds. Thus, it was concluded that scaffolds with HA-containing polymeric composites enhanced the formation of new bone tissue with increased osteoblast adhesion, osteointegration, and Ca deposition on its surface [8]. Previous reports and our preliminary results have demonstrated that HA is an important factor for cell adhesion, proliferation, and differentiation [9], [10]. Similarly, another study on the osteogenic potential of PLLA/HA/collagen scaffolds using 293 T cells and rabbit bone marrow stem cells under growth factor induction has been reported [11], [12]. Some studies have also indicated the osteoinduction property of scaffold under BMP2 stimulation, and some have reported that n-HA deposition on the PLLA/Col nanofibers is a promising strategy for early cell capture [13].

Although the use of high amount of HA (40–60%) has been found to significantly enhance fetal osteoblast cell adhesion within few days of seeding, cell proliferation within the first week has been noted to be inhibited on the mineralized scaffold. Furthermore, the high Ca content has been observed to hinder cellular activities [8]. Therefore, in the present study, a biocomposite fibrous scaffold was fabricated using 8% and 12% Col and -HA, respectively, and was subsequently blended with high-molecular-weight PLLA with an aim to produce an ideal scaffold that could elicit an optimal osteogenic response. In addition, the fabricated PLLA/HA/Col scaffold was compared with PLLA/HA and PLLA/Col with reference to chemical composition, morphology, and potential to induce osteogenic differentiation of human mesenchymal stromal cells (hMSCs) in the absence of supplemental osteogenic medium were also examined.

## Materials and Methods

### Fabrication of electrospun scaffolds

Hybrid electrospun scaffold sheets were prepared using high-molecular-weight poly(L-lactide) (PL18, Purac, The Netherlands) solutions containing HA nanoparticles (Sigma-Aldrich, USA) and/or bovine collagen (Type I) (Sigma-Aldrich, USA), with an average fiber diameter of 200–950 nm. The electrospun scaffolds were fabricated using the following solution compositions: For PLLA/Col/HA (100∶10∶15) scaffold, 2.4 g of PLLA+240 mg of collagen (Bovine Achilles Tendon) in hexafluoroisopropanol (HFIP) (Sigma-Aldrich, USA) (total weight: 30 g) and 360 mg of HA (200-nm particles) were used; for PLLA/Col (100∶10) scaffold, 2.4 g of PLLA+240 mg of collagen (Bovine Achilles Tendon) in HFIP (total weight: 30 g) were used; and for PLLA/HA (100∶15) scaffold, 2.4 g of PLLA in HFIP (total weight: 30 g) and 336 mg of HA (200-nm particles) were used. In each case, 5 ml of the solution was electrospun onto an aluminum-foil-covered, earthed rotating mandrel collector (rotation speed = 60 rpm) in an environmentally controlled cabinet (temperature = 25°C and relative humidity = 25%).

### hMSC culture

The hMSCs were isolated using previously described method [9]. The cells were cultured in medium (Invitrogen, Carlsbad, CA, USA) supplemented with 15% fetal bovine serum (FBS, Invitrogen), 100 U/mL penicillin (Sigma-Aldrich, USA), and 100 mg/mL streptomycin (Sigma-Aldrich) in tissue culture flasks at 37°C in a humidified atmosphere of 5% CO_2_. When the cells reached near confluence (80%–90%), they were detached by trypsin/EDTA (Cell Applications, San Diego, CA, USA) and then subcultured into the next passage. All cells used in this study were from a control donor and were kept in continuous cultures without any re-cryopreservation until they reached predetermined passages.

### Osteogenic differentiation of hMSCs

Prior to cell seeding, the scaffolds were treated with 70% ethanol for 15 min, and rinsed thrice in phosphate buffered saline (PBS) and once in growth medium. Scanning electron microscopy (SEM) and EDX analyses were performed before and after sterilization to monitor the changes in the fibrous scaffolds. Subsequently, the hMSCs until passage 4 were detached and seeded onto the scaffolds in 24- or 6-well plates with a cell density of 10^4^ or 10^5^/cm^2^, respectively. The medium was changed thrice at predetermined time points (0, 7, 14, and 21 days), and the samples were collected. The cell morphology, cell attachment, osteocalcin (OC) activity, matrix mineralization, immunolocalization of ECM, osteoblastic gene expression, and ultrastructural features of hMSCs during differentiation were examined to determine the potential of the scaffolds in inducing osteogenic differentiation of hMSCs.

### Characterization

The resulting scaffolds were analyzed using SEM (Phenom G2 Pro equipped with Fiber metric-Pro-Suite application) to determine the fiber diameter and its distribution. High beam of charged particles of approx. 10–30 kV was focused on the specimens using EDX (INCA Energy 200, Oxford Inst.). The number and energy of the X-rays emitted from these specimens were measured by an energy-dispersive spectrometer using Si (Li) crystal detector, and the EDX spectrum was plotted by employing Microanalysis Suite software (version 4.05-Oxford Inst.). For Fourier transform infrared spectroscopy (FTIR) analysis, the scaffolds were crushed and pressed to obtain a thin circular wafer, and the transmission spectra were recorded at the range of 4000–400/cm. The X-ray diffraction (XRD) patterns of the biocomposite scaffolds were recorded on a D8 Advance X-Ray Diffractometer (Bruker-AXS, USA) using Ni-filtered monochromatized Cu K_α_ radiation at 40 kV, 40 mA, and 25°C. The diffractogram values of PLLA, HA, and Col were obtained from the PDF-4 database (COMBICAT, University of Malaya). Following critical point drying of the samples, atomic force microscopy (AFM, NT-MDT Slover Next, Russia) with a feedback gain below 1 and 1.00-Hz probe frequency was used. The surface topography and three-dimensional (3D) images were read using Nova Px 3.2.5 software.

### Fluorescence microscopy and confocal microscopy

The scaffolds seeded with hMSCs were stained with Hoechst 33342 blue (Invitrogen, USA) and analyzed using fluorescence microscopy (Nikon C-HGFi, Japan) after 20 min of incubation at room temperature. For confocal microscopy (Leica TCS-SP5 II, Leica Microsystem, Mannheim, Germany), the post-fixed (2.5% formalin) scaffold samples were subjected to dual staining using Hoechst dye and acridine orange. The 3D image obtained from the incorporation of multiple series of images collected by confocal laser further assisted in the investigation of cell infiltration up to 0–80 µm into the scaffolds.

### Mineralization

Alizarin red (AR) S staining and Ca quantification were employed to monitor the level of mineralization at different time points (0, 7, 14, and 21 days). The scaffolds loaded with cells were fixed with 95% ethanol for 10 min, and were subsequently washed with sterile water and incubated with 0.1% AR S Tris-HCl solution at 37°C for 30 min. Then, 10 visual fields were randomly selected for data analysis. The conditioned medium was collected at different time points, and Ca quantification was performed using Quantichrom calcium assay kit. A calibration curve was obtained by reading the 96-well plates at 612 nm, and the Ca levels were calculated based on the standard linear equation and the concentration was expressed in mg/dL. It must be noted that EDTA and other Ca chelators were not used in this procedure.

### OC assay

Immunoenzymatic assay for in vitro measurement of intact human OC was performed using a commercial ELISA kit (IBL International, Germany). Briefly, the assay method used monoclonal antibody directed against epitopes of human OC. The calibrators and samples reacted with the capture monoclonal antibody coated onto the microtiter well and with a monoclonal antibody labeled with horse radish peroxidase (HRP). After the incubation period that allowed formation of sandwich, the plates were washed and chromogenic solution (TMB substrate) was added to the plates and incubated. The reaction was stopped using the stop solution provided in the kit, and the substrate turnover was read at 450 nm using a 96-well plate reader (Biotek, USA).

### Immunocytochemistry

For immunofluorescence staining, the cells on each substrate were fixed with 4% (w/v) paraformaldehyde (PFA) (Sigma) in 1×PBS for 15 min at room temperature. To permeabilize the cells, 0.1% (v/v) Triton-X 100 (Sigma) in 1×PBS was added for 5 min and washed thrice with PBS. To block nonspecific binding, the cells were incubated with 2% (v/v) goat serum (Sigma) in 1×PBS for 30 min at room temperature and washed thrice with PBS. Subsequently, the cells were incubated with primary antibodies at 4°C for 3 h. The following primary antibodies anti-fibronectin antibody [IST-9] was used for staining (1∶1000; Abcam, England). After incubation with primary antibodies, the cells were washed thrice with 1×PBS for 5 min each. Then, chicken polyclonal secondary antibody to mouse IgG – H & L (FITC) ab6810 (1∶500; Abcam, England) and 1×PBS were added for double-staining and incubated for 1 h at room temperature. The cell nuclei were counterstained using Hoechst dye. The fluorescence signals were observed under a fluorescence microscope (Nikon C-HGFi, Japan), and image analysis was carried out using NIS-elemental imaging software.

### Real-time PCR

To quantify the levels of gene expression, the total RNA was extracted from hMSCs cultured on the substrates (n = 6) at different time points (0, 7, 14, and 21 days) using the RNeasy Mini kit (Qiagen, Chatsworth, CA, USA). The concentration of the harvested RNA was determined by measuring the absorbance at 260 nm using a Nanophotometer (Implen, Germany). The first-strand cDNA was synthesized with 25 ng of pure RNA using the Superscript III First Strand Synthesis Kit, according to the manufacturer’s instructions. To examine the extent of osteogenic differentiation, quantitative real-time PCR (qRT-PCR) was performed using a StepOnePlus Real-Time PCR System (Applied Biosystems, Foster City, CA, USA) with SYBR green qPCR gene expression assays for osteogenic and chondrogenic genes. The relative expression levels of the target genes were determined using the comparative Ct method by normalization to the endogenous reference (glyceraldehyde 3-phosphate dehydrogenase) [34]. The relative gene expression involved in osteogenic and chondrogenic differentiation of hMSCs cultured on each substrate was normalized to osteogenesis markers in hMSCs cultured on the control substrate. The forward and reverse primers used for the experiment are shown in Table 1. The values obtained were averaged and expressed as means ± standard deviation (SD). Statistical differences were determined using SPSS version 10, post-hoc analysis, followed by ANOVA and LSD. The differences were considered statistically significant if the value of P was <0.05.

**Table 1:** Forward (F) and reverse (R) primers of genes.

Name	Sequence	Length
β-catenin F	TGTGGTCACCTGTGCAGCTGGA	22
β-catenin R	TGGCAGGCTCAGTGATGTCTTCC	23
Frizzled F	TGGCGCTCAGCTCGGTGGAC	20
Frizzled R	AGCGGATGCGGAAGAGGGACAC	22
PP2A F	GAGGGCCCAATGTGTGATCTGTT	23
PP2A R	CAAGCTGGTGGGCACGAGAAA	21
Col 1 F	CCCGCAGGCTCCTCCCAG	18
Col 1R	AAGCCCGGATCTGCCCTATTTAT	23
BSP F	AGGCATAAACGGCACCAGTACCA	23
BSP R	CTTGCCCTGCCTTCCGGTCT	20
OPN F	CAGCCAGGACTCCATTGACTCGA	23
OPN R	CCACACTATCACCTCGGCCATCA	23
OPN F	GTCCCCACAGTAGACACATATG	22
OPN R	TCAACTCCTCGCTTTCCATG	20
BGLAP F	GGAGGGCAGCGAGGTAGTGAAGA	23
BGLAP R	GCCTCCTGAAAGCCGATGTGGT	22
RUNX2 F	CCGCCATGCACCACCACCT	19
RUNX2 R	CTGGGCCACTGCTGAGGAATTT	22
BMP2 F	TGGCCCACTTGGAGGAGAAACA	22
BMP2 R	CGCTGTTTGTGTTTGGCTTGACG	23
Integrin F	TGGGCGCTACTGTCATTTGGG	21
Integrin R	CTGGCATCGGGTAGCTAGAGGC	22
ALP F	GATGTGGAGTATGAGAGTGACG	22
ALP R	GGTCAAGGGTCAGGAGTTC	19
Osteonectin F	TTGCAATGGGCCACATACCT	20
Osteonectin R	GGGCCAATCTCTCCTACTGC	20
C-Fos F	CGTGCCAGACATGGACCTAT	20
C-Fos R	CGGGGTAGGTGAAGACGAAG	20

## Results and Discussion

### Characterization

**Figure 1:**
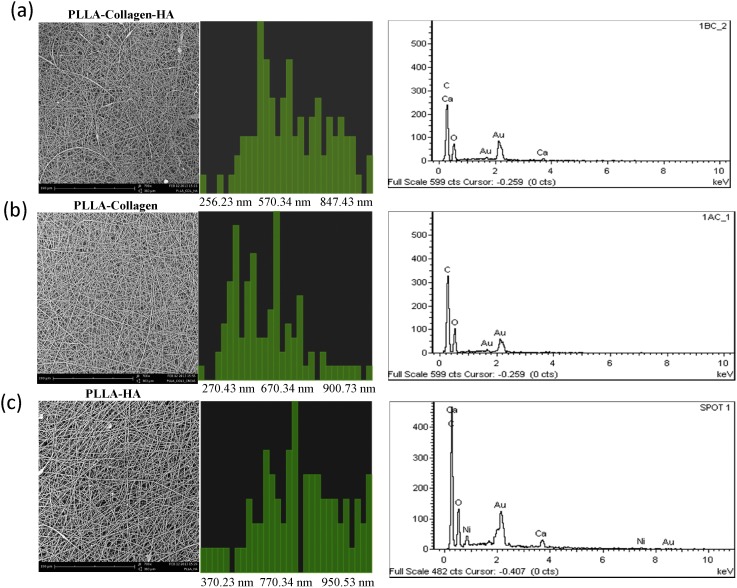
SEM, fiber diameter, and EDS of PLLA/Col/HA (a), PLLA/Col (b), and PLLA/HA (c) scaffolds. The electrospun scaffolds were subjected to surface topography analysis using SEM (Phenom G2 Pro equipped with Fiber metric-Pro-Suite application). High beam of charged particles of approx. 10–30 kV was focused on the specimens using EDX (INCA Energy 200, Oxford Inst.). The number and energy of the X-rays emitted from these specimens were measured by an energy-dispersive spectrometer using Si (Li) crystal detector, and the EDX spectrum was plotted by employing Microanalysis Suite software (version 4.05-Oxford Inst.).

**Figure 2:**
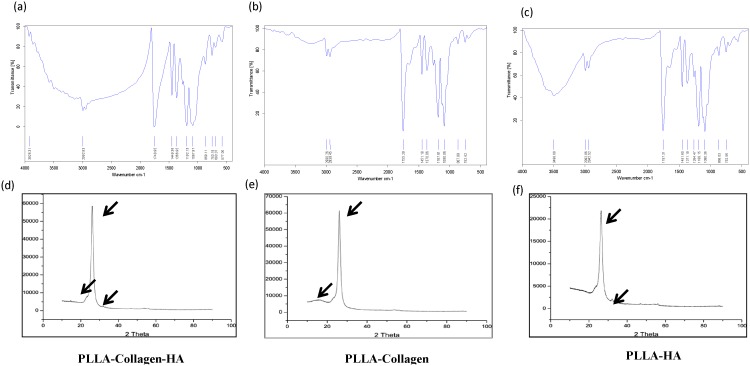
FTIR spectroscopy and XRD of the scaffolds. The scaffolds were crushed and pressed to obtain a thin circular wafer, and the transmission spectra were recorded at the range of 4000–400/cm using FTIR (a, b, and c). The XRD patterns were recorded on a D8 Advance X-Ray Diffractometer (Bruker-AXS, USA) using Ni-filtered monochromatized Cu K_α_ radiation at 40 kV, 40 mA, and 25°C. The arrows represent the characteristic peaks for individual components (PLLA, HA, Col) in electrospun nanofibrous scaffolds (d, e, and f).

The surface topography, roughness, functional groups, and elemental and phase purity of the electrospun biocomposite scaffolds were examined using SEM, AFM, FTIR, EDX, and XRD, respectively. The SEM micrographs (**Figure 1A, B, and C**) confirmed the surface topography of the fibers, and the randomly oriented fibers of PLLA/Col/HA, PLLA/Col, and PLLA/HA scaffolds had the diameter range of 256–750±100–120 nm, 270–900±89–70 nm, and 370–950±49–30 nm, respectively. It has been reported that increased polarity of the materials facilitates formation of electrospun fibers with smaller diameters, which could provide an environment similar to ECM for cell signaling and nutrient exchange [10]. Elemental analysis of the PLLA/Col/HA and PLLA/HA scaffolds confirmed the presence of elements such as Ca, O, Ni, and Au, whereas Ca was not observed in PLLA/Col scaffold (**Figures 1A, B, and C**). The FTIR (cm^−1^) results of the scaffolds are shown in **Figure 2A, B, and C**. The FTIR spectrum of PLLA/Col/HA fibers showed the characteristic peaks for PLLA, Col, and HA. The typical bands at 1087 and 1192 cm^−1^ (C-O stretching vibration), 1366 and 1449 cm^−1^ (-CH bending vibration and -CH_3_ stretching vibration, respectively), 1748 cm^−1^ (C = O stretching vibration), and 2939 and 2992 cm^−1^ (-CH stretching vibration and -CH_3_ stretching vibration, respectively) are the characteristic peaks of PLLA, which were commonly found in all three spectra. Moreover, the peaks at 753 and 869 cm^−1^ could be attributed to symmetric and asymmetric stretching of C-C_l_ bonds (respectively) in the solvent. In addition, most of the characteristic peaks of Col such as C = O stretching (amide I) and N-H bending (amide II), C-N stretching, and C-H stretching were observed to shift and coincide with the peaks of PLLA at 1748, 1087, and 2992 cm^−1^, respectively. This superposition of peaks could be caused by the interaction of PLLA and Col with HA, leading to the distinguishable broadening of the mentioned peaks and increase in their absorbance intensity, when compared with that of PLLA/Col and PLLA/HA fibers. It is also worth mentioning that the peak at 3926 cm^−1^ could be attributed to the stretching vibration of N-H groups in Col. Furthermore, the bands at 577 and 693 cm^−1^ could be assigned to the bending vibration of P-O bonds in HA, which, along with the peak at 3926 cm^−1^ (O-H stretching vibration), proved the presence of HA in the fiber composite. These peaks were shared between the FTIR spectra of two other samples (according to their composition) only with a slight shift.

**Figure 3:**
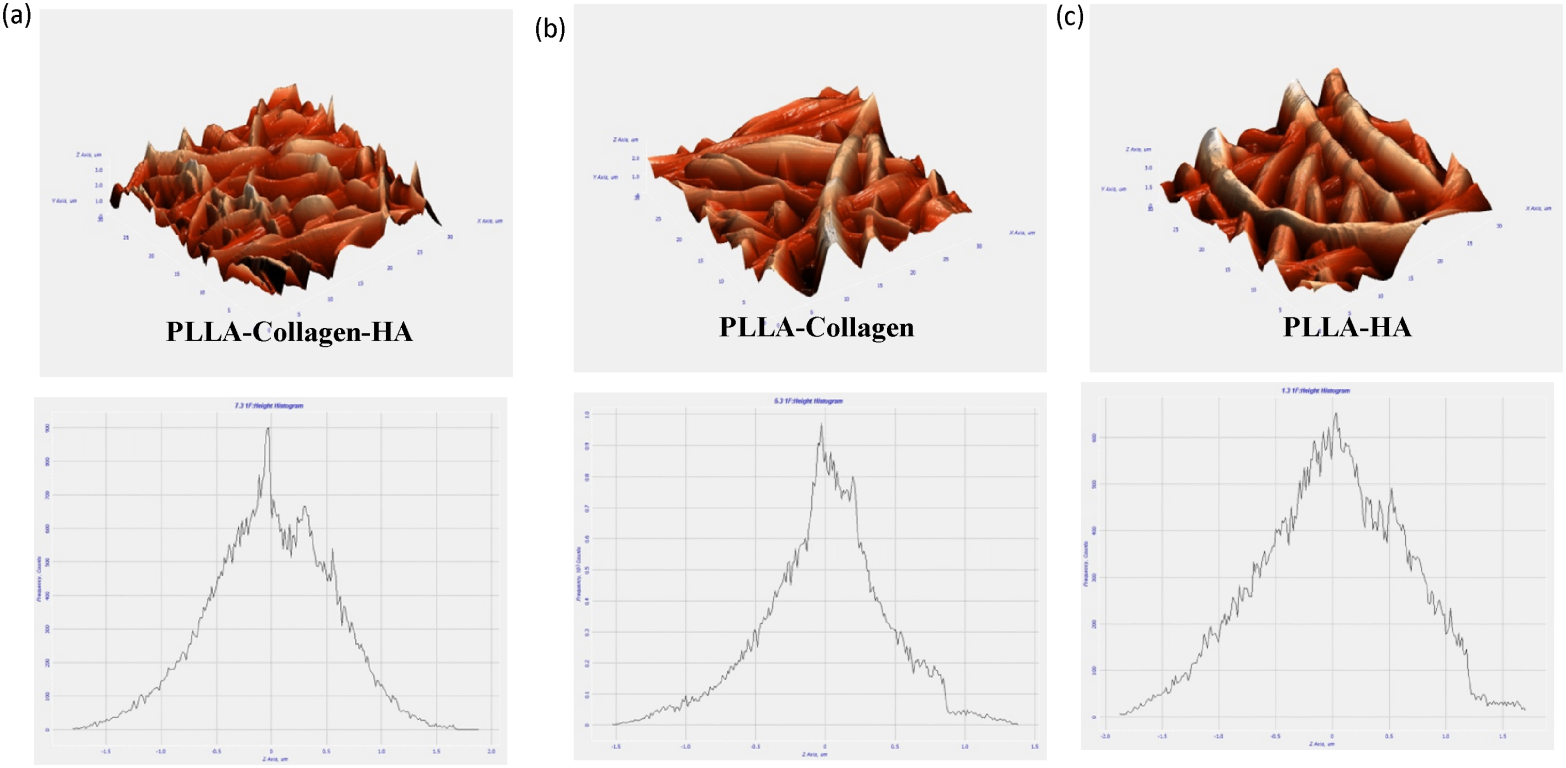
AFM of PLLA/Col/HA (a), PLLA/Col (b), and PLLA/HA (c). Following critical point drying of the samples, AFM (NT-MDT Slover Next, Russia) with a feedback gain below 1 and 1.00-Hz probe frequency was used. The surface topography and 3D images were obtained and their graph (surface roughness) was read using Nova Px 3.2.5 software.

The XRD patterns of the fiber membrane were obtained at 0–100 θ. In the range of 20–30 θ, the XRD patterns showed the broad peaks of PLLA, whereas the peaks at 30–35 θ represented the diffraction of HA (**Figure 2D, E, and F**). Previous reports have shown that the diffraction of HA may be considered as the evidence for the presence of the crystalline form of HA [14]. Some weak peaks of HA were observed for the PLLA/Col/HA and PLLA/HA scaffolds, which confirmed the dispersion nature of HA in the fibrous scaffold, rather than on the surface. Furthermore, a broad diffuse peak at about 20 θ, attributed to Col, was observed for the PLLA/Col/HA and PLLA/Col scaffolds. In this experiment, the scaffolds were not sintered to remove the organic content, because the sintering process involves high temperature, which may result in changes in the mineral phase. The distinct peak close to 32 θ for the electrospun scaffolds confirmed the presence calcium phosphate, which was absent in the PLLA/Col scaffold, but observed in the PLLA/Col/HA scaffold. This finding indicated that a mixture of calcium phosphate phases was present in the scaffold samples. **Figure 3A, B, and C** shows the AFM texture of the fiber arrangement and the mesh work pattern of the scaffolds. The representative 3D images of the non-woven fibers indicate that the surface topography was not similar. Incorporation of HA or Col into PLLA resulted in spiky ridges and patterns of grooves on the surface. Although the PLLA/Col scaffold showed some spiky ridges and groves, it was randomly arranged. The root mean square (RMS–calculated based on spectra) and average surface roughness of the PLLA/Col/HA scaffold were 0.555 and 0.443 µm, respectively; on the other hand, those values for the PLLA/Col and PLLA/HA scaffolds were 0.440 and 0.337 µm and 0.643 and 0.518 µm, respectively.

**Figure 4:**
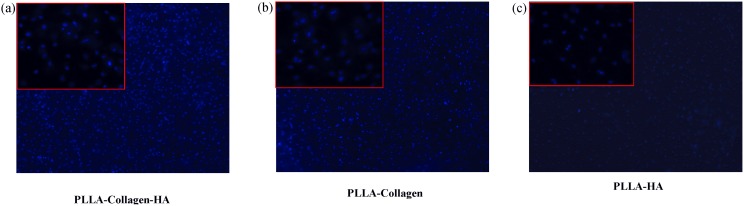
Fluorescence microscopy of cells attached to the scaffold on Day 21. The PLLA/Col/HA, PLLA/Col, and PLLA/HA scaffold were washed with ice-cold PBS twice and fixed with 4% formalin. The post-fixed samples were stained with Hoechst blue and viewed under fluorescence microscopy. The blue dots indicate the DNA of live cells stained with Hoechst blue.

Although a number of previous studies have reported on the in vitro biocompatibility of the electrospun scaffolds using cell culture, most of them have used different immortal cell lines [8], [16]–[17]. Herein, we tested the potential of the fabricated scaffolds to induce differentiation of human bone marrow stromal cells towards osteogenic lineage without supplementation of growth factors. **Figure 4A, B, and C** shows the fluorescence microscopy images (21 days) of the hMSCs cultured on the fabricated scaffolds. Hoechst staining method revealed the adherence of the cells on the scaffold surface. The blue stained cells (Day 21) were observed on all the scaffolds, which indicated that the composition of the scaffolds provided physiological environment for cell attachment and, therefore, was biocompatible. In particular, although only one representative photograph had been included, the cell density noted on the PLLA scaffold containing 12% and 8% HA and Col, respectively, was considerably better in terms of cell adhesion, when compared with that observed on the PLLA/Col and PLLA/HA scaffolds. These results confirmed that the presence of 8% and 12% Col and HA could induce cell proliferation without any growth factor supplementation. However, one limitation of the cell density result was the formation of clumps of cell population on the fibrous scaffold, which were apparently difficult to isolate using trypsinization procedure. As a consequence of this limitation, the approximate cell count information could not be obtained as that calculated in normal cell flask. In a previous study, it has been reported that the presence of collagen in the scaffolds increased their hydrophilicity and enhanced cell attachment and proliferation, while HA improved their tensile properties [18]. These results support the conclusion of the present study that hMSCs grew well on the composite scaffolds, when compared with those on pure PLLA scaffolds with either Col or HA alone.

### In vitro mineralization

**Figure 5:**
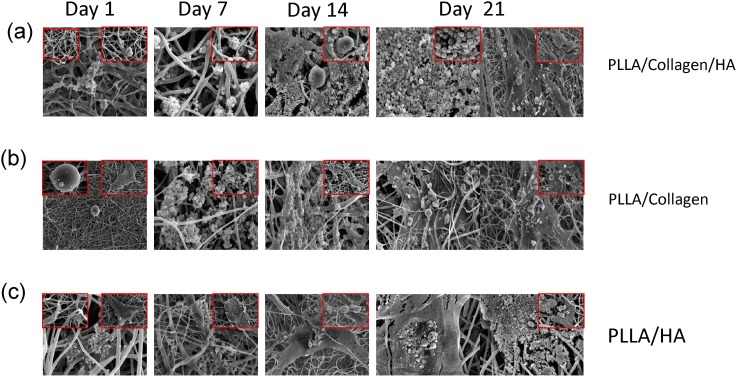
SEM of PLLA/Col/HA, PLLA/Col and PLLA/HA scaffold at different time points (0, 7, 14, and 21 days). Mesenchymal stromal cells were cultured on these scaffolds and subjected to CPD procedure. After CPD procedure, the samples were viewed under SEM (Phenom G2 Pro equipped with Fiber metric-Pro-Suite application). Arrows indicate hMSCs interaction with fibrous substrate and calcium-like apatite.

In previous studies, it has been reported that nanoscale features such as surface roughness and topography of the scaffold promote cell behavior, and that SEM images show the morphology of the cells in the differentiation phase [15]. In the present study, the SEM micrographs revealed that the hMSCs were successfully attached onto all the scaffolds examined (**Figure 5A, B, and C**). Furthermore, cell spreading on the fibrous region with characteristics such as pseudopodia was noted. In addition, greater cell spreading was observed on the PLLA/Col/HA scaffold. Morphological observation of the hMSCs grown on different scaffolds showed mineral deposition on the surface of osteoblast-like cells on the PLLA/Col/HA scaffold on Days 14 and 21, which was relatively higher than that observed on the PLLA/HA scaffold. Similar to previous studies, the PLLA scaffolds examined were found to induce formation of apatite-like calcium phosphate mineral on the surface of the cells in the presence of HA and Col. Furthermore, dimensional axonal bundle networks as well as spatial density distribution of the networks were superior in the PLLA/Col/HA scaffold, when compared with those in the PLLA/HA and PLLA/Col scaffolds. The cultured hMSCs formed an apatite-like calcium phosphate mineral and nodules on the surface of the cells. In Figure 5, the villi and cytoplasmic extensions are clearly visible, which became flattened while adhering to the surface. Moreover, the matrix vesicles secreted from the osteoblasts are also considered to be a major site of biomaterial nucleation. A previous study reported that changes in the concentration of HA in the blend can result in broken fibers [8]. Therefore, in the present study, by maintaining a constant HA amount, a weight ratio of 100∶10∶15 for PLLA:Col:HA was used; thus, the mixture contained different amounts of PLLA and Col, and uniform continuous fibers of PLLA/Col/HA were obtained. Morphological observation of the hMSCs grown on different scaffolds showed mineral deposition on the surface. The mineral deposits were significantly higher on the PLLA/Col/HA scaffold than on the PLLA/HA scaffold. The overall results suggested that electrospun PLLA/Col/HA fibrous scaffolds might more closely mimic the morphological behavior of ECM.

**Figure 6:**
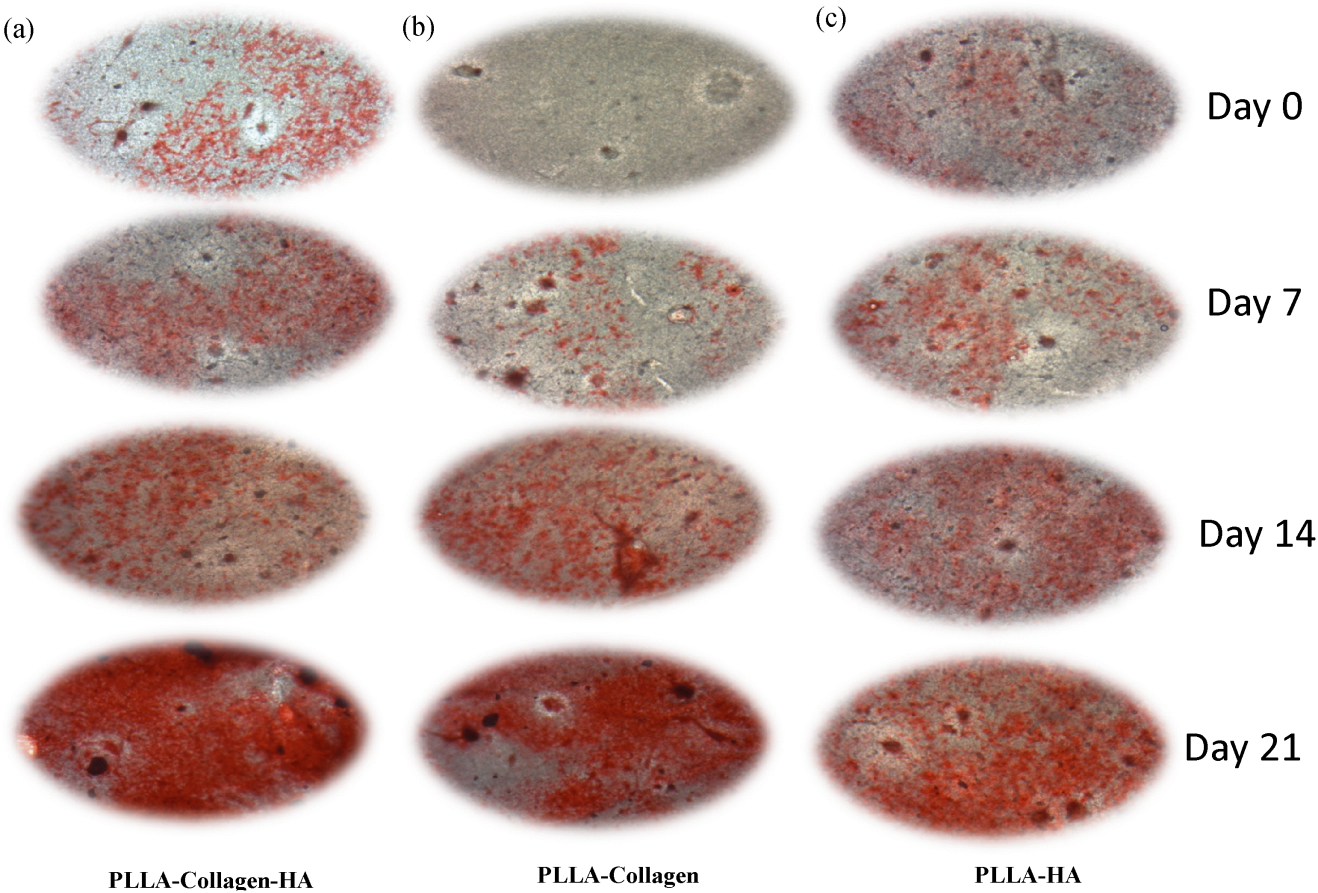
ARS staining of hMSCs on (a) PLLA/Col/HA, (b) PLLA/Col, (c) PLLA/HA fibers. The post-fixed scaffold samples from Days 0, 7, 14, and 21 were fixed with 95% ethanol for 10 min, and were subsequently washed with sterile water and incubated with 0.1% AR stain and Tris-HCl solution at 37°C for 30 min.

**Figure 7:**
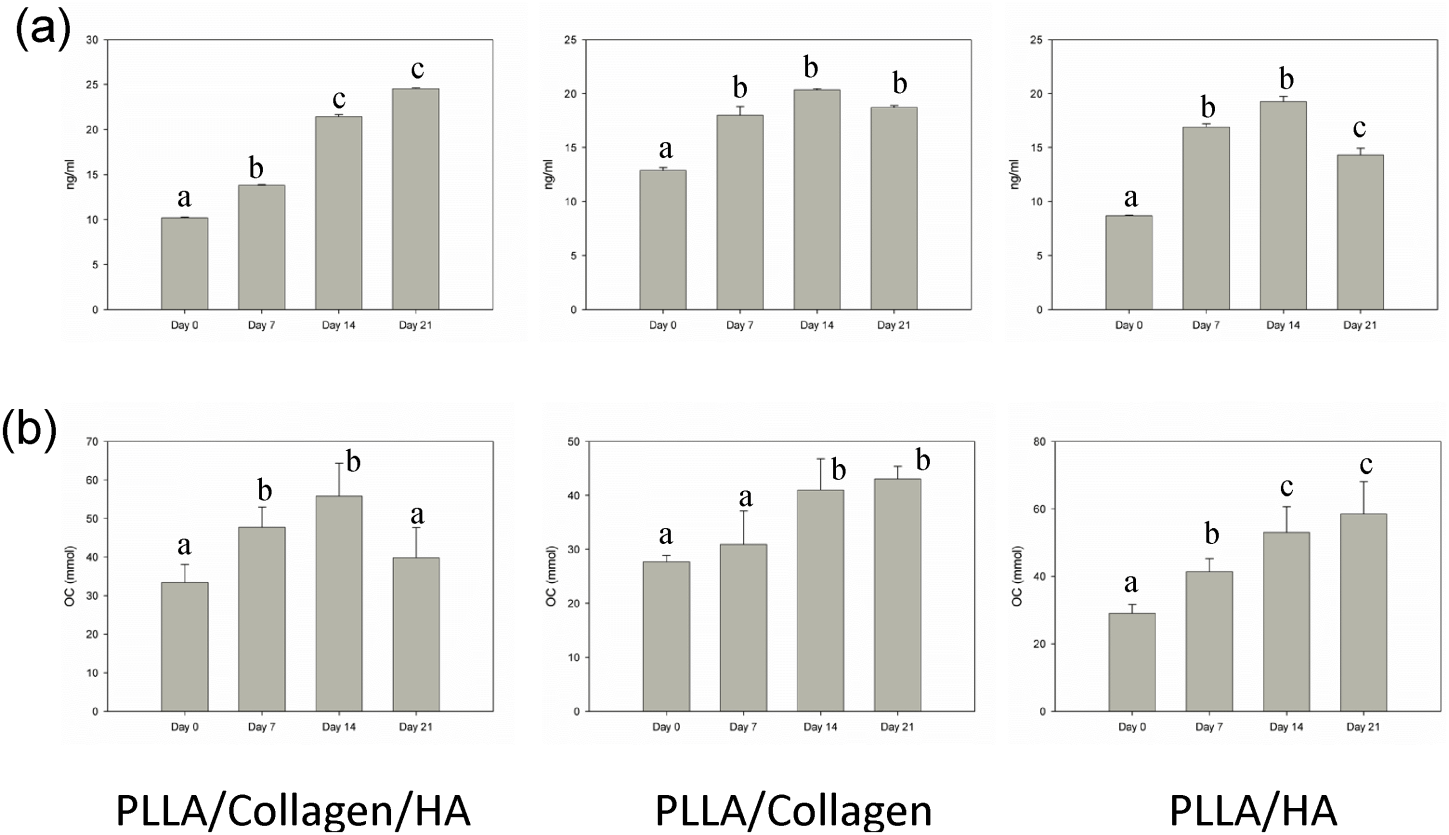
Levels of OC and Ca in PLLA/Col/HA (a), PLLA/Col (b), and PLLA/HA (c) on Days 0, 7, 14, and 21. The release of OC at different time points was quantified using commercially available ELISA kit. The conditioned medium was collected at different time points, and Ca quantification was performed using Quantichrom TM calcium assay kit. A calibration curve was obtained by reading the 96-well plates at 612 nm.

In order to confirm the mineralization capability of hMSCs, the cells were seeded onto the scaffolds and cultured for 21 days without osteogenic medium. The deposition of minerals was determined both quantitatively and qualitatively. In general, cells that undergo differentiation secrete mineral matrix; in other words, the mineralization phase involves Ca deposition and release of markers. The formation of mineral nodules in the cultures of scaffolds was examined at different time points (0, 7, 14, and 21 days) by using AR dye. The images obtained showed positive AR staining, suggesting differentiation of hMSCs into osteogenic cells with mineral deposition (**Figure 6A, B, and C**). Particularly, the intensity of AR staining was higher in the PLLA/Col/HA scaffold, when compared with that in the other two scaffolds. Qualitative results showed that the fabricated scaffold could induce Ca deposition, which is a marker for mature osteoblasts. Furthermore, in the PLLA/Col/HA scaffold, the amount of Ca deposition gradually increased (**Figure 7A, B, and C**), reaching a maximum on Day 21. Although some increase in the deposition of mineral was noted in the PLLA/Col and PLLA/HA scaffolds, the increase was relatively lower than that observed on the PLLA/Col/HA scaffold. Furthermore, Ca deposition was quantitatively significant on Day 21 in the PLLA/Col/HA scaffold, whereas it was significantly increased on Day 14 in the other two scaffolds; however, a decline in this trend was observed on Day 21. This result suggested that the ability of the scaffold to induce mineralization could be reduced. On the other hand, the increase in this trend indicated the potential of the scaffold to induce osteoblast proliferation through ECM deposition and cell-mediated early-stage mineralization. Previous studies have suggested that a threshold concentration of Ca^2+^ ions is required to stimulate subsequent activity of osteoblasts. Altogether, it can be concluded that the ability of the scaffolds to produce high Ca deposition is a good evidence for considering the use of these scaffolds in vivo [19].

To confirm the phase of osteogenic differentiation, we quantified the levels of OC after seeding the cells onto the biocomposite scaffolds. OC is a pro-osteoblastic marker that is claimed to play a significant role in modulating mineralization, because it has rich glutamic acid domains with binding affinities to both Ca and HA [20]. In the present study, the OC level increased approximately twofold in the supernatant of the cells grown on the PLLA/Col/HA scaffold on Day 14, when compared with that noted at earlier time points (**Figure 7D, E, and F**). The OC expression was higher in the 21-day samples of PLLA/Col and PLLA/HA scaffolds, when compared with that in the 14-day samples. However, this expression profile appeared to be slightly slower than those observed for the other two scaffolds. These findings correlated with the levels of Ca deposition and AR staining results. Considering the late-stage expression of OC during osteogenesis [21], it appeared that the cells had progressed along the differentiation phase before migrating into the center of the scaffolds. As OC is involved in matrix mineralization, its expression is expected to closely mirror the mineralization time course, which could be due to the differentiation of the osteoblasts and increase in the amount of later markers such as OC.

**Figure 8:**
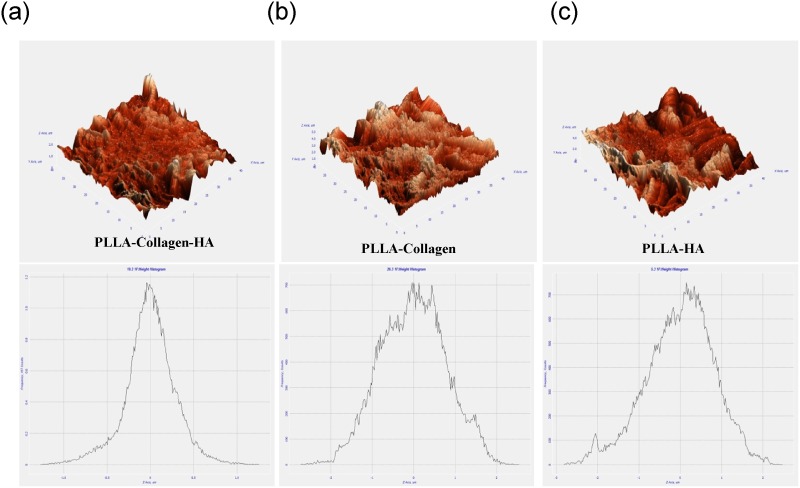
AFM of PLLA/Col/HA (a), PLLA/Col (b), and PLLA/HA (c) with cells on Day 21. Following CPD of the samples, AFM (NT-MDT Slover Next, Russia) with a feedback gain below 1 and 1.00-Hz probe frequency was used. The surface topography and 3D images were obtained and their graph (surface roughness) was read using Nova Px 3.2.5 software.

The AFM images of hMSCs on the scaffolds showed that the cells were scattered and round in appearance. Previous studies have suggested that the rounded appearance of cells could be due to the actin bundles or stress fibers [22]. An interesting observation in the present study was the roughness of the material, which was notably changed after cell growth; particularly, the RMS and average roughness after cell seeding were decreased by twofold in the case of PLLA/Col/HA scaffold (0.293 and 0.218 µm, respectively), when compared with those in the case of other scaffolds (**Figure 8A, B, and C**). On the other hand, the RMS and surface roughness of the PLLA/Col scaffold were increased by twofold (0.788 and 0.638 µm, respectively), while those of the PLLA/HA scaffold were increased to 0.819 and 0.650 µm, respectively. Previous studies have indicated that changes in the modulus of osteoblasts adhered to ECM proteins promote integrin-mediated binding, which most probably depend on fiber formation in association with focal adhesions formed at the integrin-binding sites [23].

### In vitro cell morphology and differentiation

**Figure 9:**
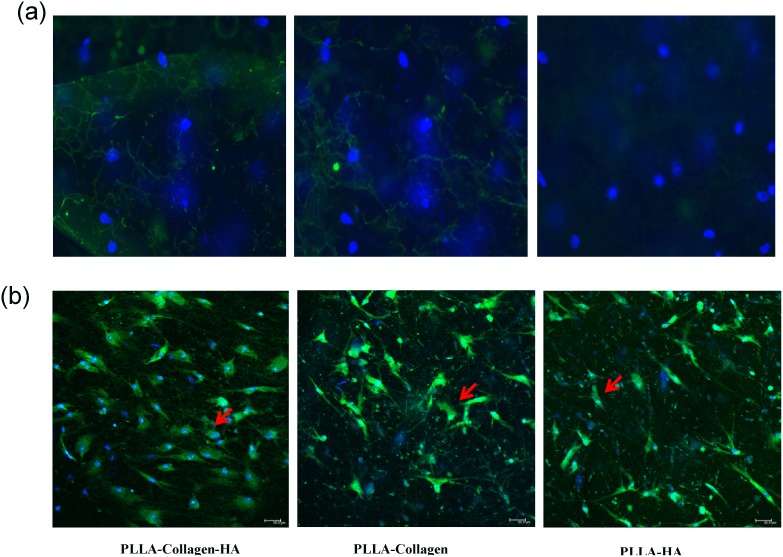
Confocal analysis and FN immunostaining representative photographs of PLLA/Col/HA (a, d, g), PLLA/Col (b, e, h), and PLLA/HA (c, f, i) after 21 days of culture. The post-fixed samples were double-stained with acridine orange and Hoechst blue and viewed using confocal microscope. The arrows indicate the green cytoplasm and blue nuclear staining of osteoblast-like cells. The post-fixed scaffold samples were immunostained with primary and secondary antibody and the fluorescence signals were observed under a fluorescence microscope (Nikon C-HGFi, Japan). The image analysis was carried out using NIS-elemental imaging software. Arrows indicate the nuclear staining (blue) and FN (green) of the ECM-like architecture after 21 days of culture.

Immunofluorescence examination showed prominent expression of fibronectin (FN) in the PLLA/Col/HA scaffold (**Figure 9A, B, and C**). FN is known to mediate cell matrix interactions and also accelerate the differentiation of progenitors into osteogenic lineage. Some reports have revealed that the distinctive difference between the rough and smooth surfaces in terms of ECM is important, despite the presence of nano-HA, which strongly binds to the FN that are ligands of integrin family of cell adhesion receptor mediators of stromal cells and osteoblasts [24]. Although the AFM images obtained in the present study are not conclusive with respect to the morphology of the osteogenic cells, our findings support the notion that positive signal in the immunofluorescence staining for FN could indicate remodeling of the cytoskeleton to increase the apparent modulus of hMSCs to osteogenic lineage. Previous studies have shown that one ECM protein, namely, FN, could provide information about the osteoblasts during their differentiation. FN, a heterodimeric glycoprotein, has cell- and matrix-binding domains, which regulate the osteogenic differentiation process through two classes of transmembrane receptors such as integrins. Osteoblasts interact with the central cell-binding domain of endogenously produced FN during the early stages of differentiation, and these interactions regulate both normal morphogenesis and gene expression [25]. In the present study, although the fluorescence signal for FN was observed in all the scaffolds, FN was predominantly localized extracellularly and was associated with cell outlines in cultures grown on the PLLA/Col/HA scaffold. Previous studies have reported that protein–phosphate composite layers containing FN can promote osteogenic activity of hMSCs by improving osteoconduction especially on the surface of HA [26]. Therefore, the results of the present study confirm that FN expression could be due to the presence of HA in the biocomposite scaffold.

Confocal microscopy was carried out using dual-staining method (Acridine orange/Hoechst blue), in which cells associated with the initial layered nodule-like formation exhibited cytoplasmic appearance with stained nuclei. In the PLLA/Col/HA scaffold, the cells exhibited definite architecture with cytoplasmic and nuclear region (**Figure 9D, E, and F**). However, the cell architecture in the other two scaffolds appeared to be altered, and the density of the cells was reduced. It must be noted that it is not easy to achieve a shift from proliferative to differentiative phase in the cells under cell-culture conditions and that the role of ECM in driving cell differentiation is not yet clear. As a result, various scaffold surfaces behave differently based on the in vitro physiological conditions and cell types. Since few years, efforts have been taken to fabricate an artificial surface mimicking natural ECM; however, not all fabricated materials are biomimetic in nature, because the currently available methodologies are not efficient in pointing out a differentiated cell using imaging studies [27]. Although previous studies have reported on the differentiation potential of the biocomposite scaffolds, the ratio of composite materials, such as Col and HA, used in the present study is different from those employed earlier [8]. Accordingly, in the current study, the presence of Col in the scaffold, with which the hMSCs had close contact, appeared to be a suitable factor that aided in the induction of differentiation of these cells along the osteogenic pathway. In the other scaffolds examined, matrix mineralization did not always occur throughout the entire scaffold by Day 21. As full scaffold penetration had not even occurred at earlier time points, one cannot expect complete mineralization to occur within a week. These results clearly demonstrated that mineralization is a slow process, and that with adequate time, the entire scaffold would be mineralized. In order to enhance the differentiation potential, we fabricated PLLA/Col fibers by incorporating 15 parts of HA using electrospinning technique to test whether the incorporation of multi-nanoporous HA could work synergistically. HA has been extensively used as an implant material in tissue engineering applications owing to its osteoinductive property as well as increased surface area and charge ratio to promote cell response, proliferation, and differentiation potential with or without any specific growth medium [27]. Although a number of the above-discussed parameters demonstrated the differentiation potential of the scaffolds examined in the present study, certain parameters, including extracellular proteins such as FN, justify our proposition that HA with PLLA/Col could enhance the differentiation phase. Osteogenesis involves the recruitment of mesenchymal cells to the osteoblast lineage and their progressive differentiation to produce a mineralized ECM. Structural ECM proteins, cytokines sequestered in the ECM, and ECM-degrading proteases and their inhibitors are all potential regulators of osteoblast differentiation and function. The observation indicating that the production and composition of ECM are carefully modulated during osteoblastic differentiation [28] suggests an important regulatory role for osteoblasts in ECM interactions. However, the functional significance of these interactions is poorly understood.

### Gene expression associated with hMSCs differentiation

The primary idea of biomimetic approach is to control and fabricate the morphology and composition of the developed biomaterials in which the crystallites of the inorganic compounds are dispersed with preferential orientation in the organic matrices [29]. Although both Col and nano-HA have the potential to mimic natural ECM, there has been particular focus on determining the appropriate percentage of HA, whose crystallite structure is similar to that of inorganic compounds in natural bone. Previous studies have examined the osteoconductivity, biocompatibility, and bioactivity of biocomposites; however, most of them have used specific growth factor to monitor osteogenic or chondrogenic differentiation [30]. The present study aimed to test whether the biocomposite has the ability to induce differentiation of hMSCs into osteogenic lineage without specific growth factors. To confirm the differentiation phase, an array of gene expression was examined at different time points (7, 14, and 21 days). The zero time point was considered as baseline, and the levels of all genes of interest were normalized with the housekeeping gene and expressed in relative fold changes.

**Figure 10:**
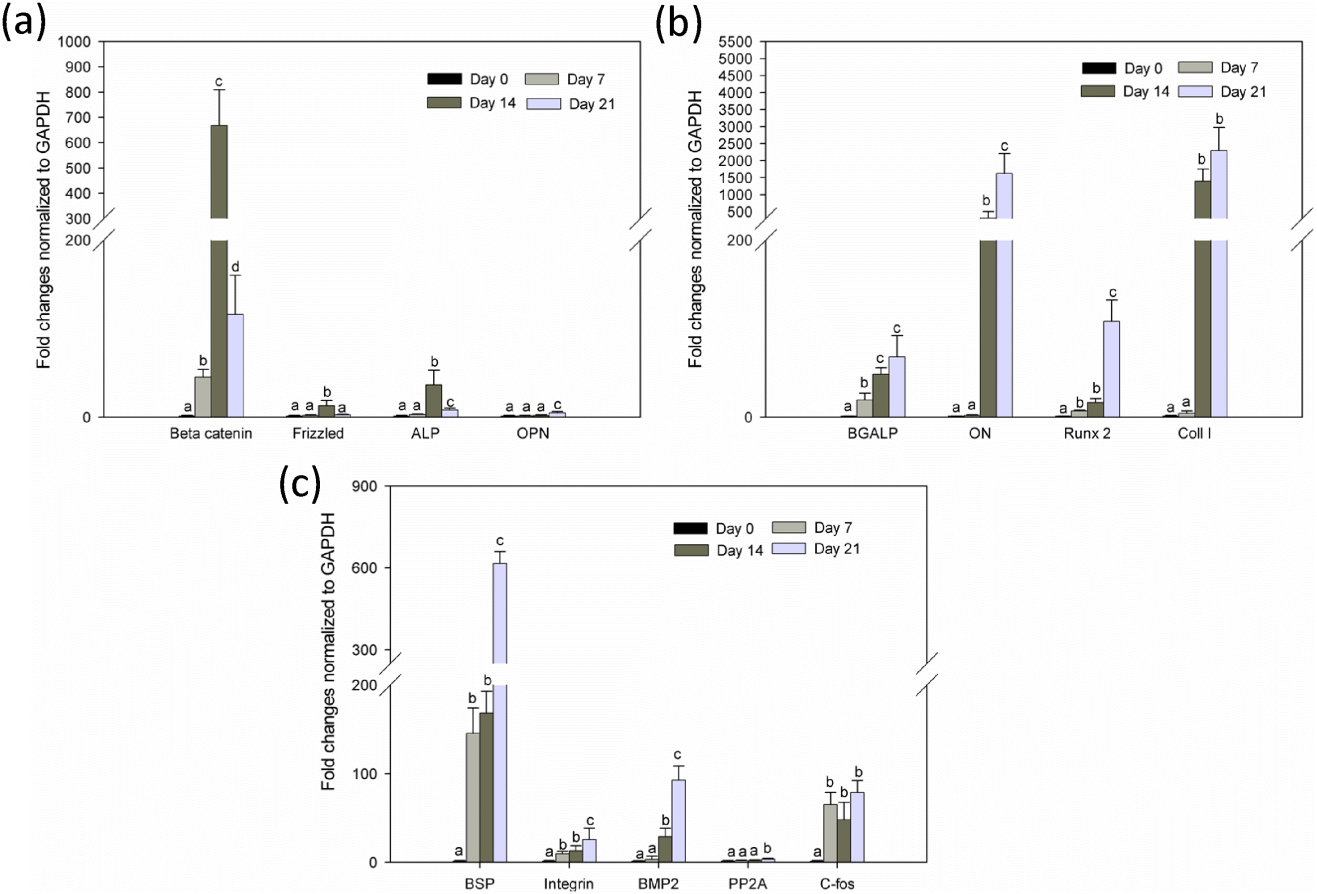
Quantitative gene expression of PLLA/Col/HA at different time points during the differentiation process. (a) Gene expression of beta-catenin, frizzled, alkaline phosphatase, and osteopontin. (b) OC (BGALP), osteonectin, Runx2, and Col. (c) Bone sialoprotein, integrin, bone morphogenic protein 2, PP2A (serine/threonine phosphatase), and c-fos. The total RNA was extracted from hMSCs cultured on the substrates (n = 6) at different time points (0, 7, 14, and 21 days) using the RNeasy Mini kit (Qiagen, Chatsworth, CA, USA). Following cDNA synthesis and qPCR, the relative gene expression was normalized to GAPDH and baseline expression. The P value was set at level <0.05 and different alphabets indicate statistically significant groups.

Earlier studies have shown that activation of beta-catenin promotes osteogenic differentiation. However, recently, a number of reports have claimed that beta-catenin signaling inhibits osteogenic differentiation and mineralization process [31]. In the present study, the PLLA/Col/HA scaffold showed significant increase (P<0.05) and decrease in beta-catenin on Days 14 and 21, respectively (**Figures 10A** **,** **11A** **, and** **12A**). A similar trend was observed in the PLLA/HA scaffold, whereas in the PLLA/Col scaffold, no decrease in the beta-catenin level was noted. This increase and decline in the beta-catenin levels indicated the balance in MSC differentiation and the fact that beta-catenin is both osteoinhibitory and inductive, but time-dependent. Studies have shown that the inhibition of MSC differentiation via beta-catenin pathway is related to the decreased expression of osteoblastic transcription factors and inhibition of other signaling cascade proteins. However, the mechanism of beta-catenin regulation of the proliferation and osteogenic differentiation of MSCs in microenvironments remains elusive. Previous studies have demonstrated that basal frizzled mRNA levels also increase when primary hMSCs are differentiated into osteoblasts, supporting the developmental regulation of the gene [32]. Accordingly, in the present study, the expression of frizzled gene was dominant on Day 14 in the PLLA/Col/HA scaffold, whereas no changes were observed in the PLLA/Col scaffold (**Figures 10A** **,** **11A** **, and** **12** **A**). However, the reason for this observation is not clear and further detailed studies are needed.

**Figure 11:**
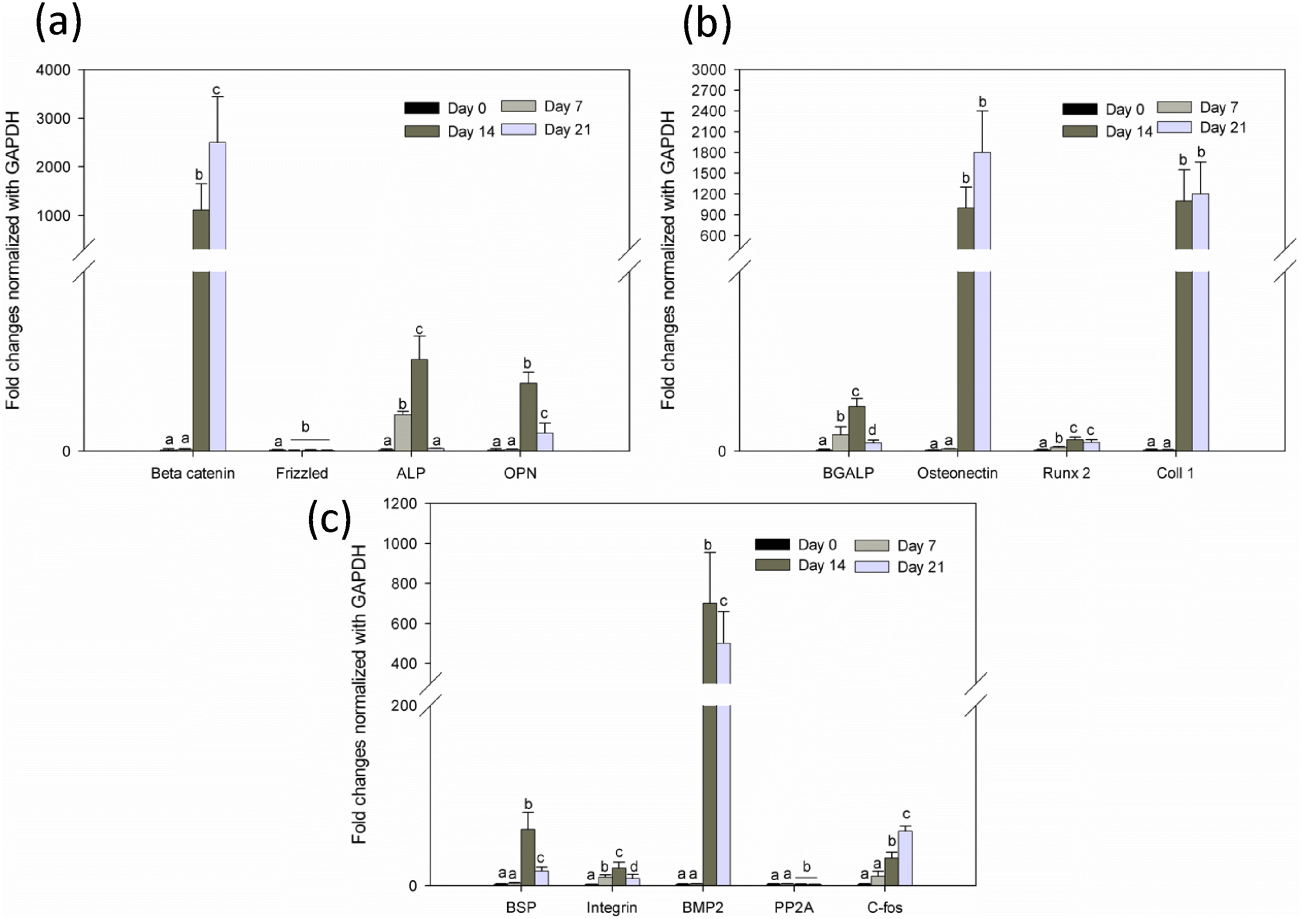
Quantitative gene expression of PLLA/Col at different time points during differentiation process. (a) Gene expression of beta-catenin, frizzled, alkaline phosphatase and osteopontin. (b) OC (BGALP), osteonectin, Runx2, and Col. (c) Bone sialoprotein, integrin, bone morphogenic protein 2, PP2A (serine/threonine phosphatase), and c-fos. The total RNA was extracted from hMSCs cultured on the substrates (n = 6) at different time points (0, 7, 14, and 21 days) using the RNeasy Mini kit (Qiagen, Chatsworth, CA, USA). Following cDNA synthesis and qPCR, the relative gene expression was normalized to GAPDH and baseline expression. The P value was set at level <0.05 and different alphabets indicate statistically significant groups.

**Figure 12:**
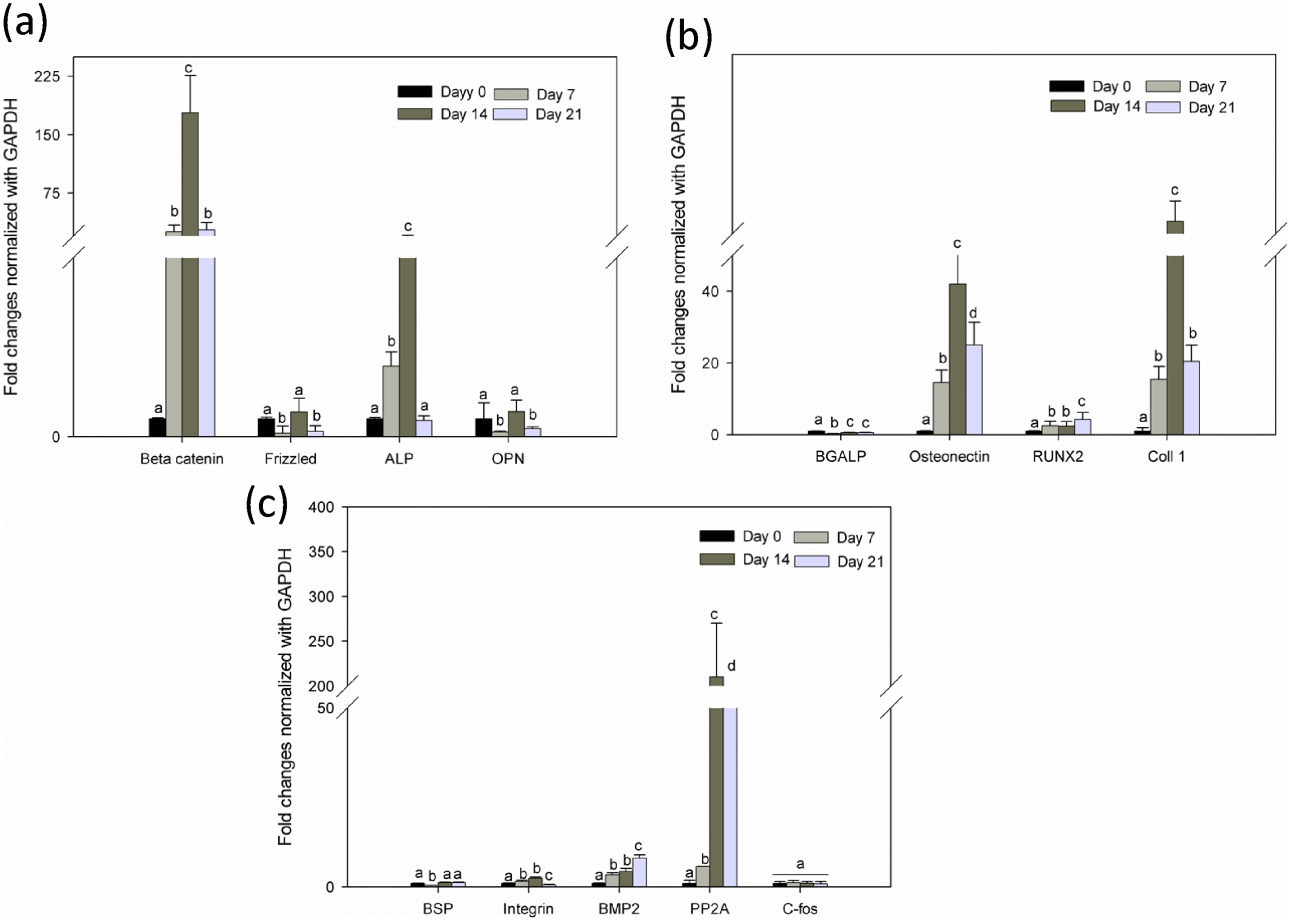
Quantitative gene expression of PLLA/HA at different time points during differentiation process. (a) Gene expression of beta-catenin, frizzled, alkaline phosphatase, and osteopontin. (b) OC (BGALP), osteonectin, Runx2, and Col. (c) Bone sialoprotein, integrin, bone morphogenic protein 2, PP2A (serine/threonine phosphatase), and c-fos. The total RNA was extracted from hMSCs cultured on the substrates (n = 6) at different time points (0, 7, 14, and 21 days) using the RNeasy Mini kit (Qiagen, Chatsworth, CA, USA). Following cDNA synthesis and qPCR, the relative gene expression was normalized to GAPDH and baseline expression. The P value was set at level <0.05 and different alphabets indicate statistically significant groups.

It has been reported that mechanical forces or topographic cues, including surface roughness and pore size (micro and nano), not only modulate cell differentiation in the presence of medium with specific growth factors, but also induce differentiation or proliferation in the absence of specific growth medium [33]. Increased expression of some osteogenic markers such as alkaline phosphatase (ALP), osteopontin (OPN), and osteocalcin (BGLAP) has (P<0.05) been reported to indicate the early osteogenic differentiation phase well supported by fibrous architecture. Nevertheless, an increase in the levels of ALP cannot be considered as the specific marker for osteogenic differentiation. To understand the osteogenic process, some lineage-specific markers and their expression during the differentiation process were examined at different time points. Among them, OC was predominantly expressed by the osteoblastic phenotype, which is a late marker during osteogenic differentiation and mineralization (Huang et al., 2007). The time course of OPN during the differentiation phase with or without extracellular molecules also correlated with the osteonectin expression. Some previous studies have indicated that the level of OPN is high during the differentiation phase [35]. In the present study, a significant increase in the level of osteonectin was observed in the PLLA/Col/HA and PLLA/Col scaffolds, whereas a decline in the osteonectin level was noted in the PLLA/HA scaffold. Nevertheless, some contrary results indicating a consistent decrease in OPN during osteogenic differentiation, both in mesenchymal cells cultured with and without other ECM proteins, such as vitronectin and Col, have been reported [36]. In the present study, the levels of OPN was significantly (P<0.05) enhanced on Day 21 in the PLLA/Col/HA scaffold, whereas the fold increase was noted on Day 14 and the expression was variable at different time points in the other two scaffolds (**Figures 10A** **,** **11A** **, and** **12A**). A previous study found that the expression of OPN increased at the beginning of osteodifferentiation and declined during 4 weeks of differentiation. Similarly, another study reported a decline in the gene expression in hMSCs after 1 week of differentiation. Likewise, in an earlier report, the gene expression in hMSCs seeded onto Col nanofibers was noted to decrease after 3 weeks, whereas in the present study, the decrease was observed after the second and third week in scaffolds containing Col. These findings indicate rapid initiation as well as termination of the differentiation process of hMSCs cultured on ECM molecules. Col is a vital protein expressed during the osteogenic differentiation process.

In the present study, the expression of collagen gene was evident during the early time point in the PLLA scaffold containing Col and HA, indicating the initial cellular response to osteoinductive factors. Similar to an earlier study, Col was significantly expressed after Day 7, which was produced extracellularly forming a matrix within the scaffold structure. This is an important step because Col is necessary for osteogenesis, which stimulates the pre-osteoblast cell surface integrin leading to the activation of other core binding factors, and contact with Col results in an increased level of surface integrin [36].

Furthermore, in correlation with the BGLAP quantification data, the post-proliferative osteoblast maker, OC, was also used to assess the differentiation progress of hMSCs on the electrospun scaffolds (**Figures 10B** **,** **11B** **, and** **12B**). The OC gene expression was found at an early time point, which could be related to the partial differentiation of the hMSCs scaffold culture. This finding was correlated with the AR staining results, which revealed that the staining was not very widespread. The BGLAP expression was more intense in the 21-day samples than that in the 14-day samples of PLLA scaffolds containing both HA and Col. However, the expression of this specific gene was not significant at the early and late time points of the differentiation phase. The difference between 14- and 21-day culture samples was marked in all cases, with substantial mineralization noted in osteoinductive factor treated scaffolds on Day 21. The elevation of BGLAP was significant on Day 14 in the PLLA/Col scaffold, when compared with that in the PLLA/Col/HA scaffold, which could be due to the increased time required for the cells to respond to the fibrous network structure and the surface of the PLLA/Col/HA scaffold. These results clearly demonstrated that mineralization is a slow process, and that with adequate time, the entire scaffold will be mineralized.

In addition to the early osteogenic marker genes, we examined the expression of Runt-related transcription factor 2 (Runx2), which is considered to be the central control gene within the osteoblast phenotype (**Figures 10B** **,** **11B** **, and** **12B**). The results obtained showed considerable increase (P<0.05) in the levels of Runx2 gene on Day 21, indicating the ability of the scaffold material to support osteogenic differentiation; however, the relative fold expression was significantly high in the PLLA/Col/HA scaffold on Day 21. The expression of Runx2 and Osterix could be found in two distinct stages of hMSCs differentiation into osteoblasts. The Runx2 can upregulate the expression of other genes, such as that encoding osteonectin, and simultaneously interact with Osterix. It has been reported that Runx2 can also downregulate genes such as those encoding Col and bone sialoprotein (BSP). Col is an important organic component of ECM produced by osteoblast cells [37]. In the present study, a decline in Col and BSP levels was not noted in the PLLA/Col/HA scaffold on Day 21, whereas increased levels of Col and an increased BSP level on Day 14 and a decreased BSP level on Day 21 were observed in the PLLA/Col scaffold (**Figures 10B** **,** **11B** **, and** **12B**). On the other hand, in the PLLA/HA scaffold, an increase in Col was noted on Day 14, whereas no significant alterations in the BSP level was observed on Days 14 and 21 (**Figures 10C** **,** **11C** **, and** **12C**). It has been reported that BSP is a mitogenic agent for preosteoblast cells, which can promote differentiation of progenitor cells to osteoblasts, ultimately stimulating bone mineralization [38]. In the present study, a gradual increase in BSP in the PLLA/Col/HA and PLLA/Col scaffolds indicated that the material may support differentiation of the hMSCs. Furthermore, the results of the PLLA/HA scaffold analysis suggested early differentiation of the cells or reduction in the number of matured cells on Day 21.

An ideal scaffold should exhibit properties such as osteoinductivity, osteoconductivity, and osteogenesis. In the present study, the increase in BMP2 was consistent in all the three scaffolds, which principally demonstrated that the material could initiate stem cells or progenitors to undergo differentiation process, particularly, to form osteoblast-like cells [39]. At an early time point, the SEM results revealed the presence of chondrogen-like cells. To confirm this observation, we examined the expression of some genes such as those encoding collagen II, SOX9, and cartilage-specific proteoglycan core protein, and found that their expression was very negligible (data not shown). The role of topography in the differentiation of the progenitors to specific lineage depends on the micro- or nanocavities or roughness, which induces the cells to express some genes during the differentiation process [21]. Although some increase in the levels of Col could be noted in the present study, it could not be considered as an osteoconductive factor. In contrast, marked osteoinduction could have occurred on the PLLA/Col/HA scaffold, when compared with that on the PLLA/Col or PLLA/HA scaffolds. In general, there exist differences in the mechanism of action of bone induction by osteoconductive biomaterials and growth factors such as BMP. Biomaterials usually exhibit bone induction through the intramembranous ossification process, whereas the growth factors induce bone formation through endochondral ossification [40]. Although the growth factors are efficient in inducing osteogenesis, osteoconductive materials are preferred for preclinical experiments. Interestingly, till date, no studies have shown any clear evidence regarding why biomimetic nanocomposites that could induce osteogenesis are not effective as growth factor supplements. The reason for this could be the proportion of the elements and the technique used to design a biomimetic nano- or microcomposite scaffolds, and the choice of the cell type, i.e. primary or immortal cells. The ratio of HA and cell type in the scaffold used in the present study is relatively different from that employed earlier. Hence, the results of this study are not correlated to the previously reported data, especially with respect to the characterization and differentiation process.

**Figures 10C** **,** **11C** **, and** **12C** show the gene expression of integrin in biocomposite scaffolds. The enhancement of cell adhesion has been reported to remarkably affect cell contraction, ligation, and intracellular signaling. In general, the fibrous surface provides another dimension that enhances cell matrix interactions and cell–cell communications, which are important for oxygen and nutrients exchange between the cells, when compared with the normal culture plate environment [41]. This surface not only improves the environment for cell attachment, but also induces ECM-like organization and differentiation abilities. The significantly enhanced integrin expression indicates the role of topography in controlling progenitors. The cell spreading on the fibrous structure could be due to the binding of the integrins on the surface to appropriate adhesion proteins on the matrix. The large surface-to-volume ratio of the scaffold provides more binding sites for the receptors for improved cellular functions [42]. In addition, the random circular nanostructures induce changes in the adhesion formation and morphology, affecting the cytoskeleton tension, which could direct osteogenic differentiation. Previous reports have shown that the cell matrix interactions are mostly dependent on integrins. The attachment of cells to ECM causes clustering of integrins into focal adhesion complexes and activates intracellular signaling cascades into the central nucleus. Earlier studies have reported that scaffolds could induce osteogenic differentiation with the supplementation of medium containing specific growth factors; however, the findings have been inconsistent. The major reason for this could be the different ratio of HA and collagen as well as the cell type used.

PP2A (serine/threonine phosphatase) regulates diverse cellular functions, predominantly transcription and signaling. Although PP2A does not have a direct role in the osteogenic differentiation process, it regulates mitogen-activated protein kinase signaling, and has been noted to dephosphorylate mitogen-activated and extracellular receptor kinase in various cell lines. As ERK activation is involved in the osteogenic differentiation of MSCs, the agents that can modulate PP2A may have a role in osteogenic differentiation [43]–[45]. In the present study, the PP2A gene expression was significant on Day 21 in the PLLA/Col/HA scaffold, whereas the expression was variable in the other two scaffolds, and the reason for these changes is not clear (**Figures 10C** **,** **11C** **, and** **12C**). Furthermore, no PP2A expression was observed in the PLLA/Col scaffold on Days 14 and 21, whereas the expression was significantly elevated on Day 14 and decreased on Day 21 in the PLLA/HA scaffold.

The level of c-fos was significantly increased in the PLLA/Col/HA scaffold, but not in the PLLA/HA scaffolds (**Figures 10C** **,** **11C** **, and** **12C**). The expression of c-fos is considered as amitogenic stimulus and its downstream effects are numerous in addition to its role in proliferation, transformation, and apoptosis. Furthermore, it has been reported that c-fos has a role in the formation of osteoblast and adipocyte lineage [46]. However, some studies have shown c-fos as a non-specific marker, because it is expressed in different cell types, including those of chondrogenic lineage [47]. From the results obtained in the present study, it was evident that the fabricated material supported adhesion and proliferation, and that 12% HA could act as a chelating agent for the mineralization of osteoblasts.

## Conclusion

Altogether, the osteoinductive potential of the biocomposite scaffold, PLLA/Col/HA, was appreciable especially in terms of marker secretion and gene expression. However, when compared with the previous reports, the results obtained in the present study are different, which could be due to the relatively diverse ratio of the concentration of elements used. The present study used hMSCs without supplementation osteogenic medium and a direct comparison was observed between PLLA scaffolds with HA or Col alone and in combination. The presence of Col and HA on the fiber surface promptly affected differentiation towards an osteogenic lineage, thus supporting the notion that composite fibers could be beneficial for stem cell based therapies in bone tissue engineering.
