# Correction: A Comparative Study on *In Vitro* Osteogenic Priming Potential of Electron Spun Scaffold PLLA/HA/Col, PLLA/HA, and PLLA/Col for Tissue Engineering Application

**DOI:** 10.1371/journal.pone.0114775

**Published:** 2014-11-25

**Authors:** The PLOS ONE Staff

There are several errors in the published article.

The fourth and fifth author’s names are spelled incorrectly. The correct names are: Malliga R. Murali and Sangeetha V. Naveen. The correct citation is: Balaji Raghavendran HR, Puvaneswary S, Talebian S, Murali MR, Naveen SV, et al. (2014) A Comparative Study on *In Vitro* Osteogenic Priming Potential of Electron Spun Scaffold PLLA/HA/Col, PLLA/HA, and PLLA/Col for Tissue Engineering Application. PLoS ONE 9(8): e104389. doi:10.1371/journal.pone.0104389

Throughout the article P < 0.05 should read p < 0.05.

In the Materials and Methods, under “Real-time PCR”, the sentence “To examine the extent of osteogenic differentiation, quantitative real-time PCR (qRT-PCR) was performed using a StepOnePlus Real-Time PCR System (Applied Biosystems, Foster City, CA, USA) with SYBR green qPCR gene expression assays for osteogenic and chondrogenic genes” should read “To examine the extent of osteogenic differentiation, quantitative real-time PCR (qRT-PCR) was performed using a StepOnePlus Real-Time PCR System (Applied Biosystems, Foster City, CA, USA) with SYBR green.”

In the Materials and Methods, under “Real-time PCR,” the sentence “The relative gene expression involved in osteogenic and chondrogenic differentiation of hMSCs cultured on each substrate was normalized to osteogenesis markers in hMSCs cultured on the control substrate” should read “The relative gene expression involved in osteogenic differentiation of hMSCs cultured on each substrate was normalized to osteogenesis markers in hMSCs cultured on the control substrate.”
